# Supplementary figures and images for: Endogenous corazonin signaling modulates the post-mating switch in behavior and physiology in females of the brown planthopper and Drosophila
Source: eLife. 2026 May 12;14:RP109297. doi: 10.7554/eLife.109297 (PMC13167112; doi:10.7554/eLife.109297)

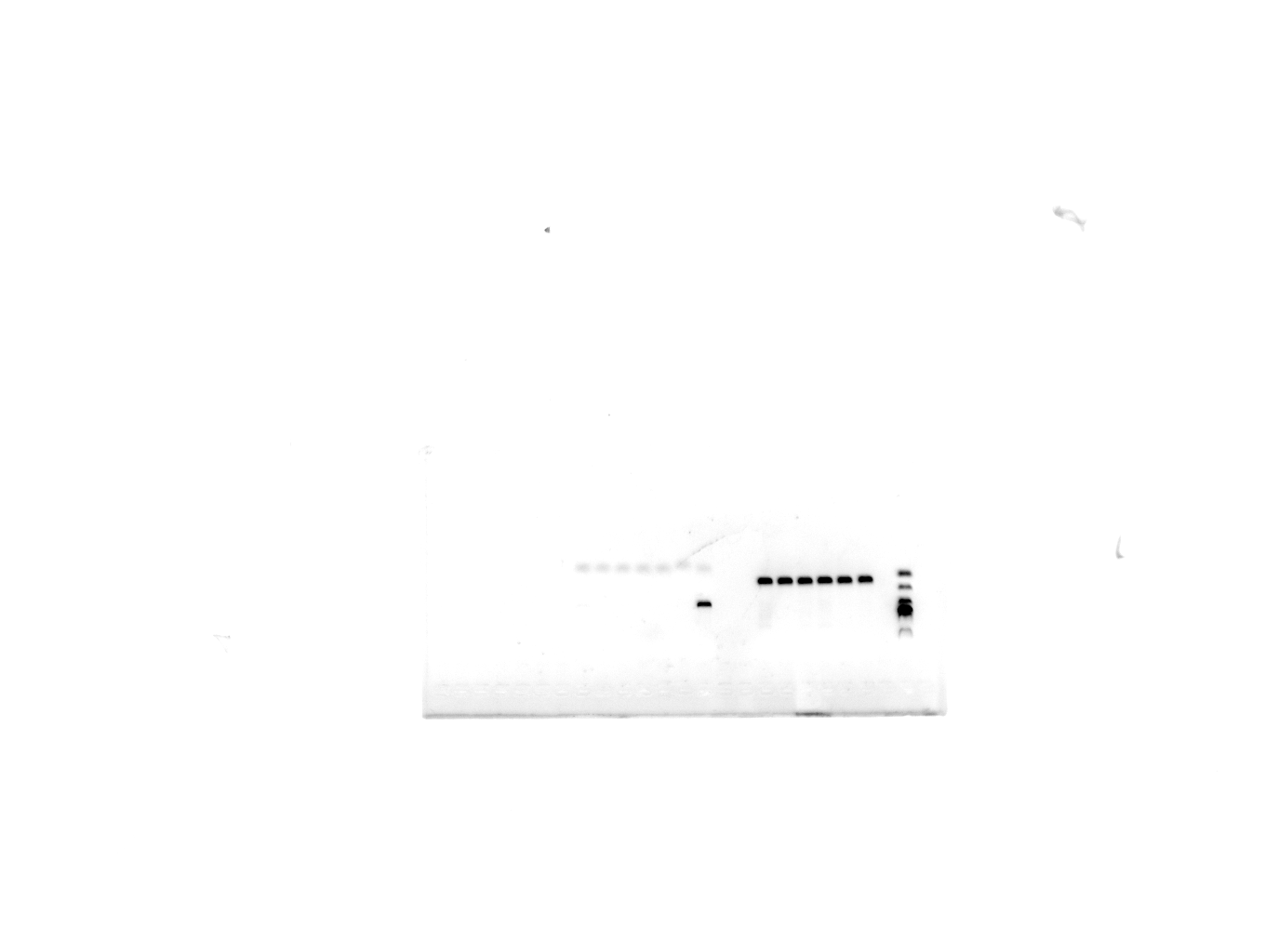

Supplement: Figure 2—source data 1. [file elife-109297-fig2-data1.zip › Raw data/Raw data.tif]

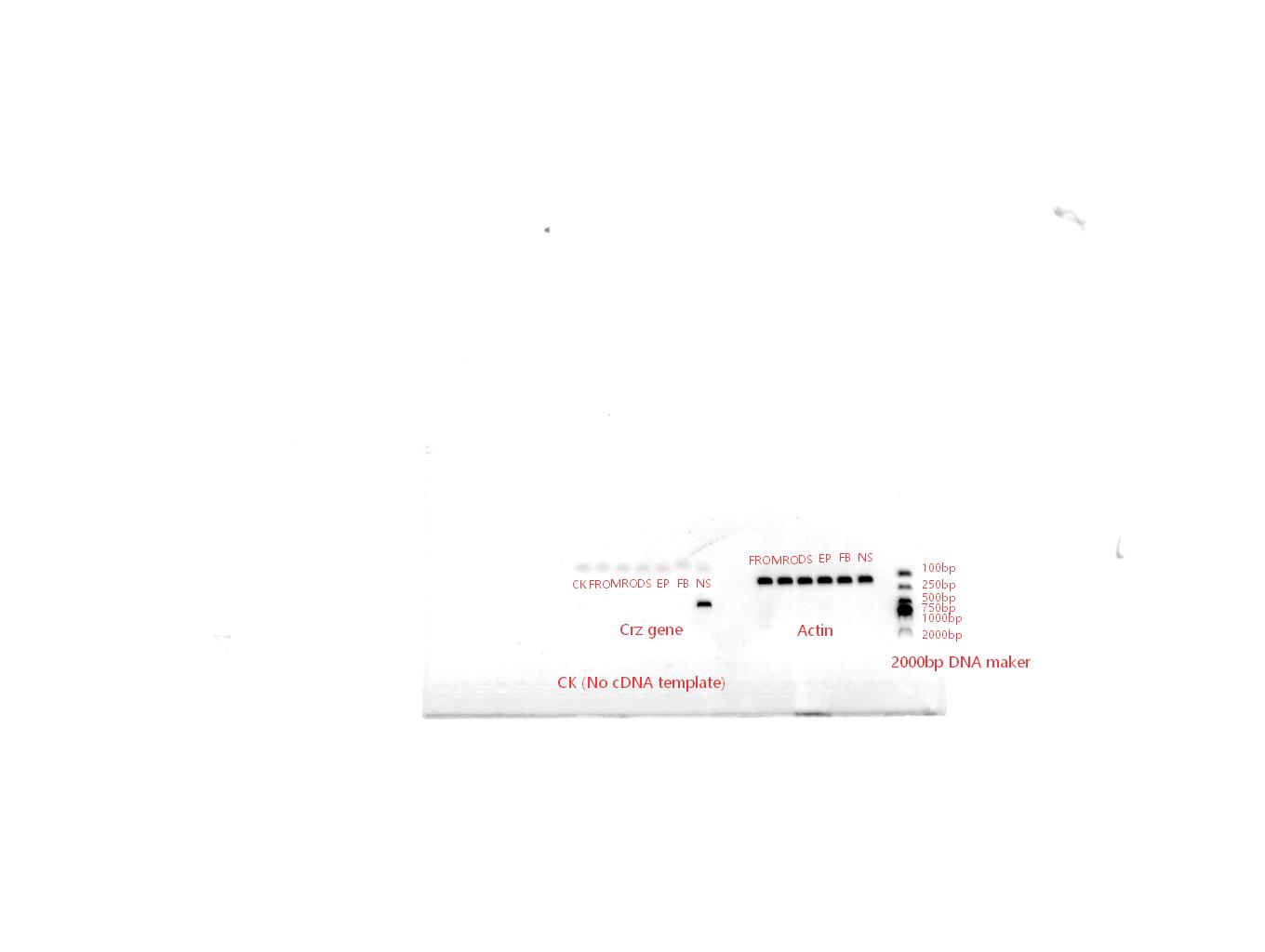

Supplement: Figure 2—source data 2. [file elife-109297-fig2-data2.zip › Raw data with indication/Raw data with indication-new.jpg]

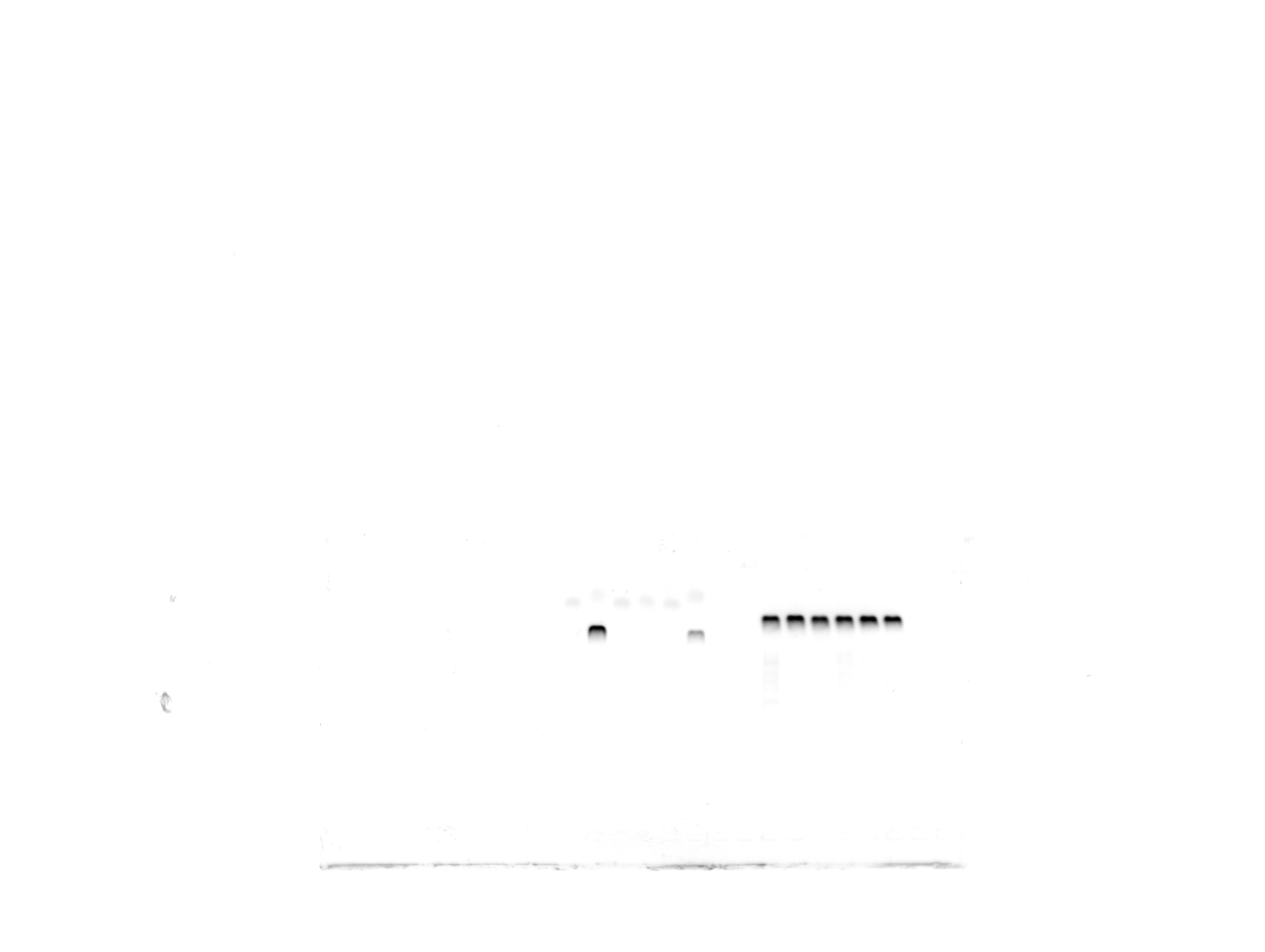

Supplement: Figure 4—source data 1. [file elife-109297-fig4-data1.zip › Raw data/Raw data.tif]

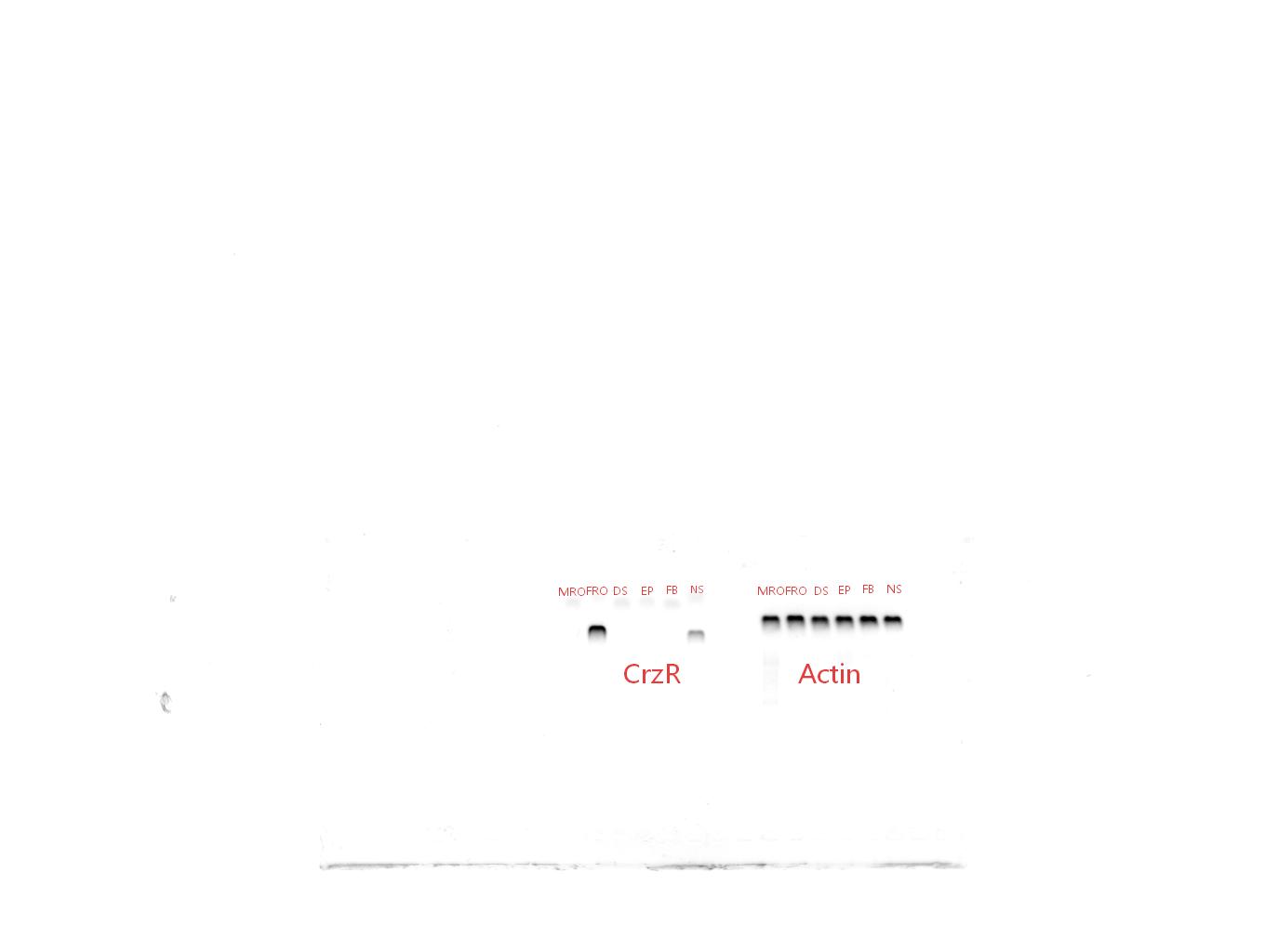

Supplement: Figure 4—source data 2. [file elife-109297-fig4-data2.zip › Raw data with indication/Raw data with indication.jpg]

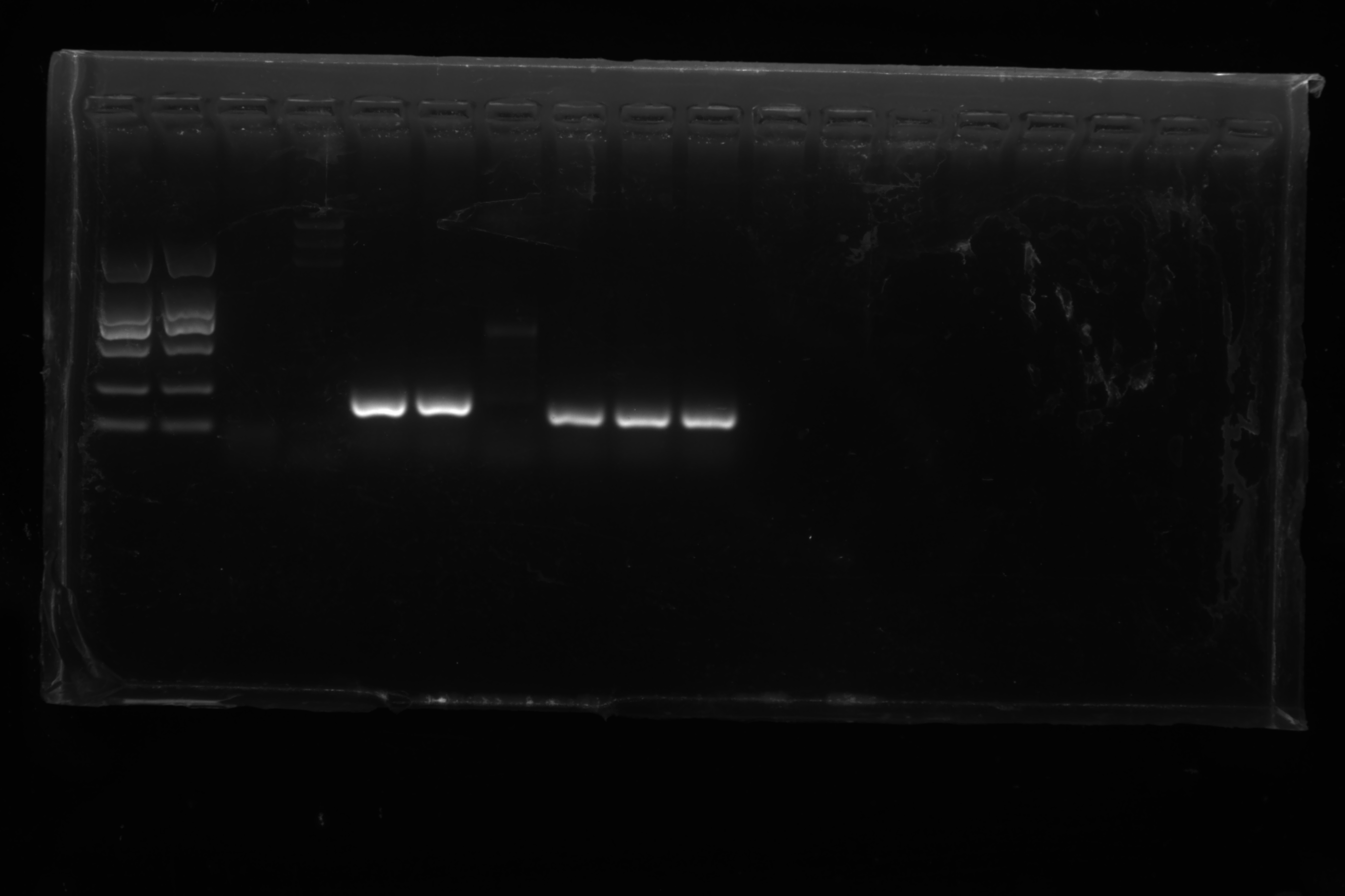

Supplement: Figure 5—source data 1. [file elife-109297-fig5-data1.zip › raw data/raw data.tif]

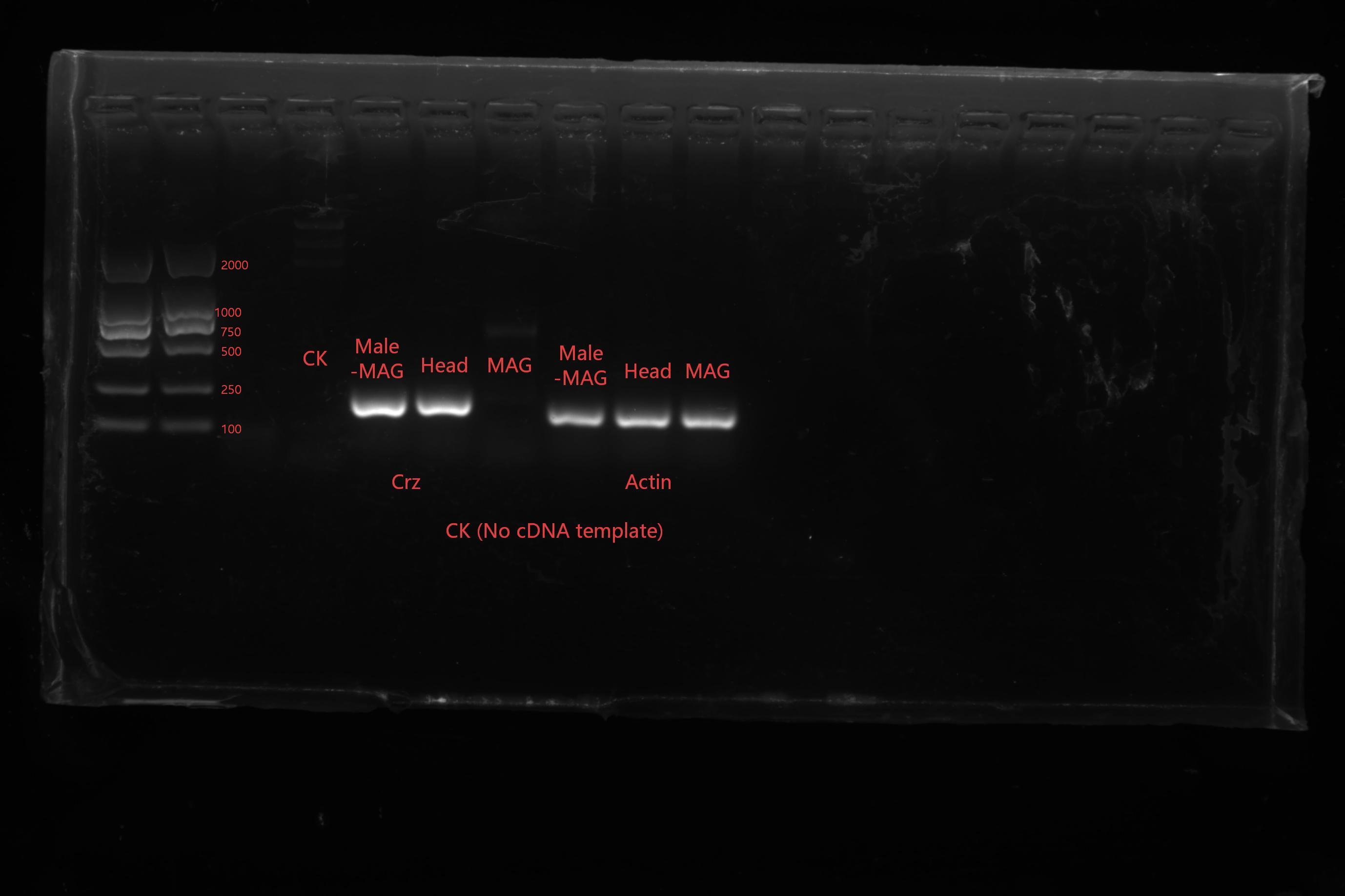

Supplement: Figure 5—source data 2. [file elife-109297-fig5-data2.zip › Raw data with indication/raw data with indication(1).jpg]
