# Supplementary material for: Endogenous corazonin signaling modulates the post-mating switch in behavior and physiology in females of the brown planthopper and Drosophila
Source: eLife. 2026 May 12;14:RP109297. doi: 10.7554/eLife.109297 (PMC13167112; doi:10.7554/eLife.109297)
Supplement: Supplementary file 1. [file elife-109297-supp1.docx]

Table legend:

**Supplementary file 1. Primers used in this study**

|  | Sequence (5'-3') | Purpose |
| --- | --- | --- |
| NlCrz-F | ACCGCATCAGGACGACAATA | Gene clone |
| NlCrz-R | ACCATAGCTGTTGAGCTACAAAT |  |
| NlCrz-RNAi-F | TAATACGACTCACTATAGGGGCTAGTGTTGCAATGCTGTTG | *dsNlCrz* synthesis |
| NlCrz-RNAi-R | TAATACGACTCACTATAGGGGCTGTTGAGCGTTAGACTGT |  |
| QNlCrz-F | AGACAGTGACCACAGCTCAA | qRT-PCR for NlCrz |
| QNlCrz-R | GAGTACTGGAACGTCTGTGC |  |
| NlCrz-sgRNA-F1 | TAATACGACTCACTATAGGCACAGGAGTGATGCAGAC | sgRNA synthesis for NlCrz knock-out |
| NlCrz-sgRNA-R1 | TTCTAGCTCTAAAACGTCTGCATCACTCCTGTGCC |  |
| NlCrz-sgRNA-F2 | TAATACGACTCACTATAGCTACTAGTGCTGGGCTGCA | sgRNA synthesis for NlCrz knock-out |
| NlCrz-sgRNA-R2 | TTCTAGCTCTAAAACTGCAGCCCAGCACTAGTAGC |  |
| NlCrz Check F | ACCATAGCTGTTGAGCTACAAAT | To analyze NlCrz mutant |
| NlCrz Check R | TGCAATGCTGTTGTCGCGAC |  |
| NlCrzR-F | AATCAGGCGATGAACCTCAGTT | Gene clone |
| NlCrzR-R | ACGCAGTTCTATGACGTGAGG |  |
| NlCrzR-RNAi-F | TAATACGACTCACTATAGGGCGCCGTCTACACACTCATCT | *dsNlCrzR* synthesis |
| NlCrzR-RNAi-R | TAATACGACTCACTATAGGGCTACCAGCTTCGTACAGCGT |  |
| QNlCrzR-F1 | CTGGTGCTAATAGGGCTGGA | qRT-PCR for NlCrzR |
| QNlCrzR-R1 | TCCGGTGTACAGTTGCTCTT |  |
| QNlCrzR-F2 | TGCTCACCATCGCCTCTATC | qRT-PCR for NlCrzR |
| QNlCrzR-R2 | TGCCATGGTTCCGTGTAGAA |  |
| NlCrzR-F1 | CCCACTAGCCAGCAACCATT | RT-PCR for NlCrzR |
| NlCrzR-R1 | TCCTCCACAAATGGTCCACG |  |
| NlCrzR-sgRNA-F1 | TAATACGACTCACTATAGGCACGCACTCGAGCTGCCT | sgRNA synthesis for NlCrzR knock-out |
| NlCrzR-sgRNA-R1 | TTCTAGCTCTAAAACAGGCAGCTCGAGTGCGTGCC |  |
| NlCrzR-sgRNA-F2 | TAATACGACTCACTATAGGCGATGGTGAGCAGGTTGC | sgRNA synthesis for NlCrzR knock-out |
| NlCrzR-sgRNA-R2 | TTCTAGCTCTAAAACGCAACCTGCTCACCATCGCC |  |
| NlCrzR Check-F | GGCAGCAACCTGAGCCACCC | To analyze NlCrzR mutant |
| NlCrzR Check-R | GTCTTACCAGCGACCCAAGC |  |
| QNlvg-F | GTGGCTCGTTCAAGGTTATGG | qRT-PCR for Nlvitellogenin |
| QNlvg-R | GCAATCTCTGGGTGCTGTTG |  |
| QNlvgR-F | AGGCAGCCACACAGATAACCGC | qRT-PCR for Nlvitellogenin receptor |
| QNlvgR-R | AGCCGCTCGCTCCAGAACATT |  |
| QNl Macc-F | TCTTTGGAAGTGGTGATTCCATCT | qRT-PCR for NlMacc |
| QNl macc-R | TGTCTACTTCACATACATGTGGCT |  |
| QNl itpl-1-F | GATCAGATTCAATTCACATTCCTC | qRT-PCR for Nlitpl-1 |
| QNl itpl-1-R | AGATTGTAGCAGTCCTCGCATA |  |
| DmCrz-F | CCTCTTCACGCTCTCCATGTG | RT-PCR for DmCrz |
| DmCrz-R | TCGGTTGGCATTGAAGTCCG |  |
| DmActin-F | TGTGACGAAGAAGTTGCTGC | RT-PCR for Actin 5C |
| DmActin-R | CTCATCACCCACGTACGAGT |  |
| QNl Actin-F | TAACGAGAGGTTCCGTTGCC | internal control for qRT-PCR |
| QNl Actin-R | GACAGGACAGTGTTGGCGTA |  |
| QNl 18sR-F | CGCTACTACCGATTGAA |  |
| QNl 18sR-R | GGAAACCTTGTTACGACTT |  |
